# Spatiotemporal Changes in the Gene Expression Spectrum of the β2 Adrenergic Receptor Signaling Pathway in the Lungs of Rhesus Monkeys

**DOI:** 10.1007/s00408-021-00420-2

**Published:** 2021-01-29

**Authors:** Zhongmei Zheng, Bangrong Cao, Yu Hu, Liang Xie, Ling Gu, Fang Shi, Hanmin Liu

**Affiliations:** 1 Department of Pediatrics, West China Second University Hospital, Sichuan University, No. 20, Section 3, South Renmin Road, Chengdu, 610041 China; 2 Department of Pediatrics, Chengdu Women’s and Children’s Central Hospital, School of Medicine, University of Electronic Science and Technology of China, Chengdu, China; 3 Radiation Oncology Key Laboratory of Sichuan Province, Sichuan Cancer Hospital & Institute, Sichuan Cancer Center, School of Medicine, University of Electronic Science and Technology of China, Chengdu, China; 4 Department of Pediatrics, Mianyang Central Hospital, Mianyang, China; 5 The Vascular Remodeling and Developmental Defects Research Unit, West China Institute of Women and Children’s Health, West China Second University Hospital, Sichuan University, Chengdu, China; 6 Key Laboratory of Birth Defects and Related Diseases of Women and Children (Sichuan University), Ministry of Education, West China Second University Hospital, Sichuan University, Chengdu, China

**Keywords:** Β2 adrenergic receptor, Gene expression, Signaling pathway, Rhesus monkeys

## Abstract

**Objective:**

β2 adrenergic receptor (ADRB2) agonists mainly participate in regulation of airway function through the ADRB2-G protein-adenylyl cyclase (AC) signaling pathway; however, the key genes associated with this pathway and the spatiotemporal changes in the expression spectrum of some of their subtypes remain unclear, resulting in an insufficient theoretical basis for formulating the dose and method of drug administration for neonates.

**Methods:**

We performed sampling at different developmental time points in rhesus monkeys, including the embryo stage, neonatal stage, and adolescence. The MiSeq platform was used for sequencing of key genes and some of their subtypes in the ADRB2 signaling pathway in lung tissues, and target gene expression was normalized and calculated according to reads per kilobase million.

**Results:**

At different lung-developmental stages, we observed expression of *phenylethanolamine N-methyltransferase* (*PNMT*), *ADRB2*, *AC, AKAP* and *EPAC* subtypes (except *AC8, AKAP4/5*), and various *phosphodiesterase* (*PDE*) subtypes (*PDE3*,* PDE4*,* PDE7*, and *PDE8*), with persistently high expression of *AC6*, *PDE4B*, and *AKAP*(1/2/*8/9/12/13*, and *EZR*) maintained throughout the lung-developmental process, *PNMT*, *ADRB2*, *AC*(4/6), *PDE4B,* and *AKAP*(1/2/*8/9/12/13, EZR,* and *MAP2*)were highly expressed at the neonatal stage.

**Conclusion:**

During normal lung development in rhesus monkeys, key genes associated with ADRB2–G protein–AC signaling and some of their subtypes are almost all expressed at the neonatal stage, suggesting that this signaling pathway plays a role in this developmental stage. Additionally, *AC6*, *PDE4B*, and *AKAP*(1/2/*8/9/12/13,* and *EZR*) showed persistently high expression during the entire lung-developmental process, which provides a reference for the development and utilization of key gene subtypes in this pathway.

## Introduction

β2 adrenergic receptor (ADRB2) agonists are currently widely used in the treatment of childhood and adult asthma and chronic obstructive pulmonary disease. ADRB2 agonists are mainly used to treat neonatal wet lung syndrome [1], bronchopulmonary dysplasia (BPD), and wheezing in premature infants [2, 3]; however, the indications are not unified. These agonists directly act on ADRB2 to activate ADRB2–G protein–adenylyl cyclase (AC) signaling associated with regulation of airway function. Therefore, receptor-density distribution and intensity are key factors affecting drug effects; however, the key genes associated with this signaling pathway and the spatiotemporal changes in the expression spectrum of some of their subtypes remain unclear. In particular, there are few studies on neonates at the perinatal stage, resulting in an insufficient theoretical basis for formulating the dose and method of drug administration for neonates.

ADRB2 is the main subtype of βAR in human lung. ADRB2 is a downstream effector of the adrenergic signaling pathway and specifically binds adrenaline synthesized by phenylethanolamine N-methyltransferase (PNMT) catalysis of noradrenaline [4]. The binding of β2AR agonist with ADRB2 on cell membrane can activate adenylate cyclase(AC), and AC catalyzes the conversion of ATP into cAMP, increasing the level of cyclic adenosine monophosphate (cAMP) to induce airway smooth muscle (ASM) relaxation [5]. There are currently at least nine AC subtypes identified in humans [6]. cAMP performs its biological actions by activating various effectors, including protein kinase A (PKA) and exchange protein directly activated by cAMP (EPAC), which has two isoforms, EPAC1 and EPAC2 [7]. cAMP and its effectors are strictly spatiotemporal controlled by a scaffold protein family of more than 50 members called A-kinase anchoring proteins (AKAPs) [8]. Meanwhile, phosphodiesterase (PDE) is a key enzyme involved in cAMP hydrolysis, resulting in a decrease in its concentration [9].

There are 11 families and 30 subtypes of PDE in humans, of which PDE4(A, B, C, and D), PDE7(A and B), and PDE8 (A and B) have high specificity for cAMP [10, 11]. PDE3(A and B) and PDE4 are the two major cAMP-hydrolyzing enzymes [12].

In this study, we measured and analyzed spatiotemporal changes in key genes intimately associated with the ADRB2–Gs–AC signaling pathway and some of their subtypes during lung development in rhesus monkeys, particularly during the neonatal stage. The findings provide a theoretical basis for rational drug use in the neonatal stage and references for the development and utilization of some key gene subtypes in this pathway.

## Materials and Methods

### Animals

Rhesus monkeys were selected as experimental animals based on their closest phylogenetic distance with humans relative to other animals (~ 25 million years). Rhesus monkeys have a life span of 30 years and a gestation period of ~ 165 days. All monkey samples and corresponding transcriptome data were obtained from previous studies [13, 14]. Briefly, the samples were divided into several different developmental stages, including the early stage of embryonic development (Day 45; *n* = 1; and Day 70, *n* = 1), the middle of embryonic development (Day 100, *n* = 3), late embryonic development (Day 137, *n* = 1; Day 157, *n* = 1; and Day 163, *n* = 1), the neonatal stage (Day 4 after birth, *n* = 1; Day 5 after birth, *n* = 1; and Day 7 after birth, *n* = 1), and adolescence (Year 5 after birth, *n* = 2; and Year 7 after birth, *n* = 1). All animal experiments complied with the ARRIVE guidelines [15]. The data sources have been published, and the ethical reviews have been declared in the original literature [14].

Sodium pentobarbital was used to anesthetize animals at the aforementioned times. Standard surgical procedures were used to extract the trachea, bronchi, and lungs of the embryos and young monkeys. For the time points of less than 100 days, the whole lung was isolated. At other time points, a lobe was isolated. The obtained samples were washed with PBS before TRIzol treatment. The experimental methods are described and cited according to the original references [13, 14].

### Sequencing

TRIzol (Thermo Fisher Scientific, Waltham, MA, USA) was used to extract total RNA according to manufacturer instructions. Total RNA quality was examined using spectrophotometry and agarose gel electrophoresis, and a commercial kit (Takara, Dalian, China) was used to synthesize cDNA, which was subsequently sequenced using the MiSeq platform (Illumina, San Diego, CA, USA). The expression levels of the target genes were normalized and calculated according to reads per kilobase million (RPKM). The sequencing part is based on the description of the original reference and cited [13, 14].

### Expression Analysis

We measured the expression levels of key genes associated with the ADRB2 signaling pathway and some of their subtypes, including *PNMT*, *ADRB2*, *AC*(*1*–*9*), *PDE3*(*A* and *B*), *PDE4*(*A, B, C*, and *D*), *PDE7*(*A* and B), *PDE8*(*A* and *B*), *AKAPs*(*1*–*14*, *EZR* and *MAP2*), and *EPAC*(*1* and *2*). Unsupervised clustering and heat maps were used to study dynamic changes in expression of these genes during lung development in rhesus monkeys.

### Statistical Analysis

The pheatmap package of R (https://www.rdocumentation.org/packages/pheatmap/versions/1.0.12/topics/pheatmap) was used to plot gene expression maps. SPSS (v.22.0; IBM Corp., Armonk, NY, USA) was used for data processing. Statistical analysis was performed using analysis of variance(ANOVA)and an independent sample *t* test, and a *P* < 0.05 was considered significant.

## Results

### Gene Expression of Key Genes Associated with the ADRB2 Signaling Pathway

**Fig. 1:**
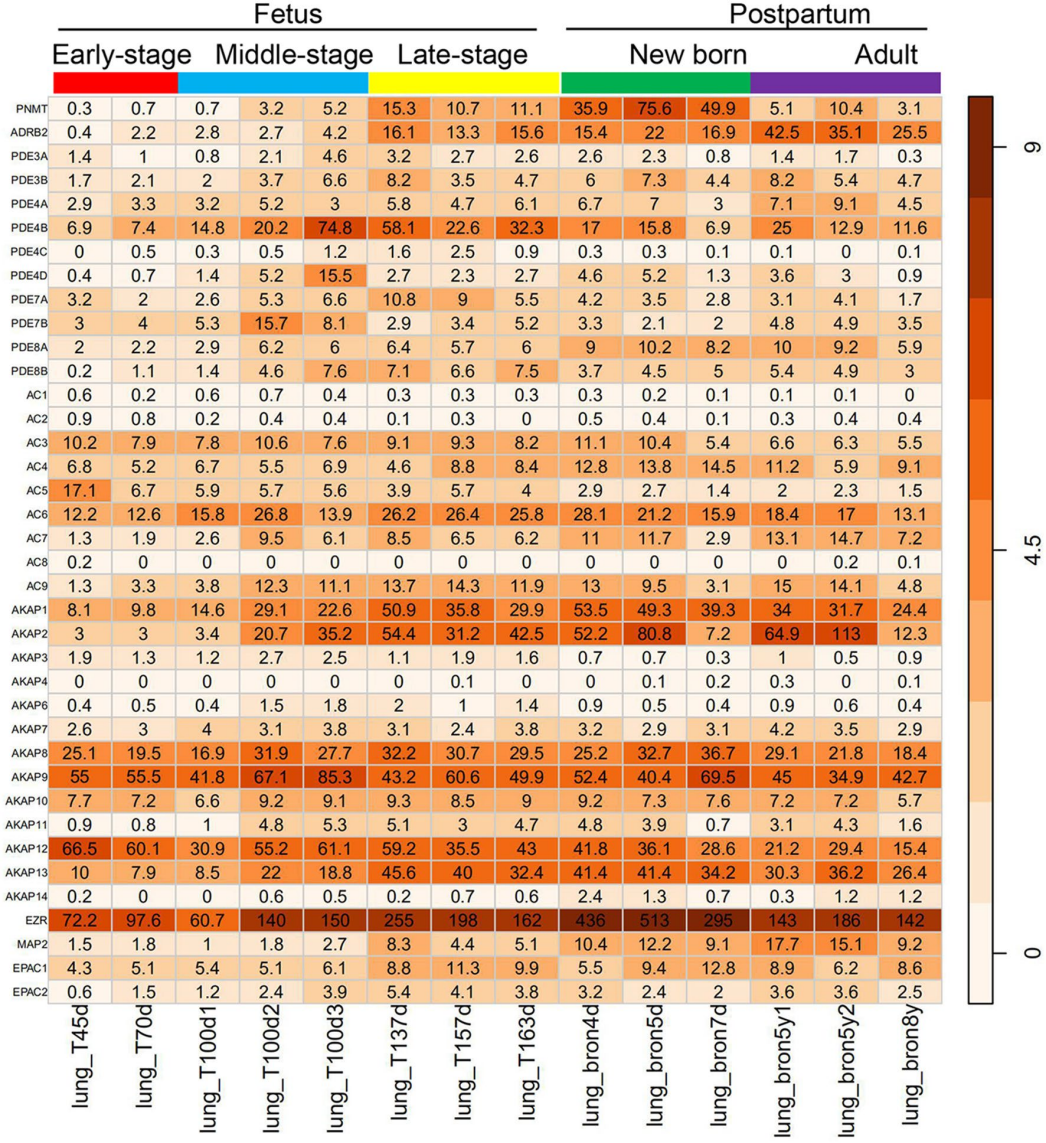
Gene expression profiles of 38 key genes associated with signaling during lung development in healthy rhesus monkeys. Each row represents a single gene, and the histogram in each row represents independent lung samples according to time. T45d and T70d represent Days 45 and 70 of gestation, respectively. T100d1, T100d2, and T100d3 represent the first, second, and third monkey on Day 100 of gestation, respectively. T137d, T157d, and T163d represent Days 137, 157, and 163 of gestation, respectively. Bron4d, bron5d, and bron7d represent Days 4, 5, and 7 after birth, respectively. Bron5y1, bron5y2, and bron8y represent the first monkey at 5 years after birth, second monkey at 5 years after birth, and a monkey at 8 years after birth, respectively. Numbers in the matrix are gene expression values (RPKM), and the color gradient represents log_2_(RPKM + 1)

The results showed that *PNMT*, *ADRB2*, all *AC, AKAP* subtypes (except *AC8, AKAP4/5*), all *PDE* subtypes (*PDE3*, *PDE4*, *PDE7*, and *PDE8*), and *EPAC* subtypes associated with the ADRB2–Gs–AC signaling pathway were expressed during the entire lung-development process. Among these genes, *AKAP5* was not detected. *AC1*, *AC2*, *PDE4C*, *AKAP6*, and *AKAP14* had extremely low expression (RPKM < 1). *AC6*, *PDE4B*, and *AKAP*(1/2/*8/9/12/13,* and *EZR*) were highly expressed (RPKM > 10) during the entire lung-development process, whereas *PNMT*, *ADRB2*, *AC*(*4/6*), *PDE4B,* and *AKAP*(1/2/*8/9/12/13, EZR,* and *MAP2*)were highly expressed (RPKM > 10) at the neonatal stage (Fig. 1).

### *PNMT* and *ADRB2* Exhibit Different Expression Profiles During Lung Development

**Fig. 2:**
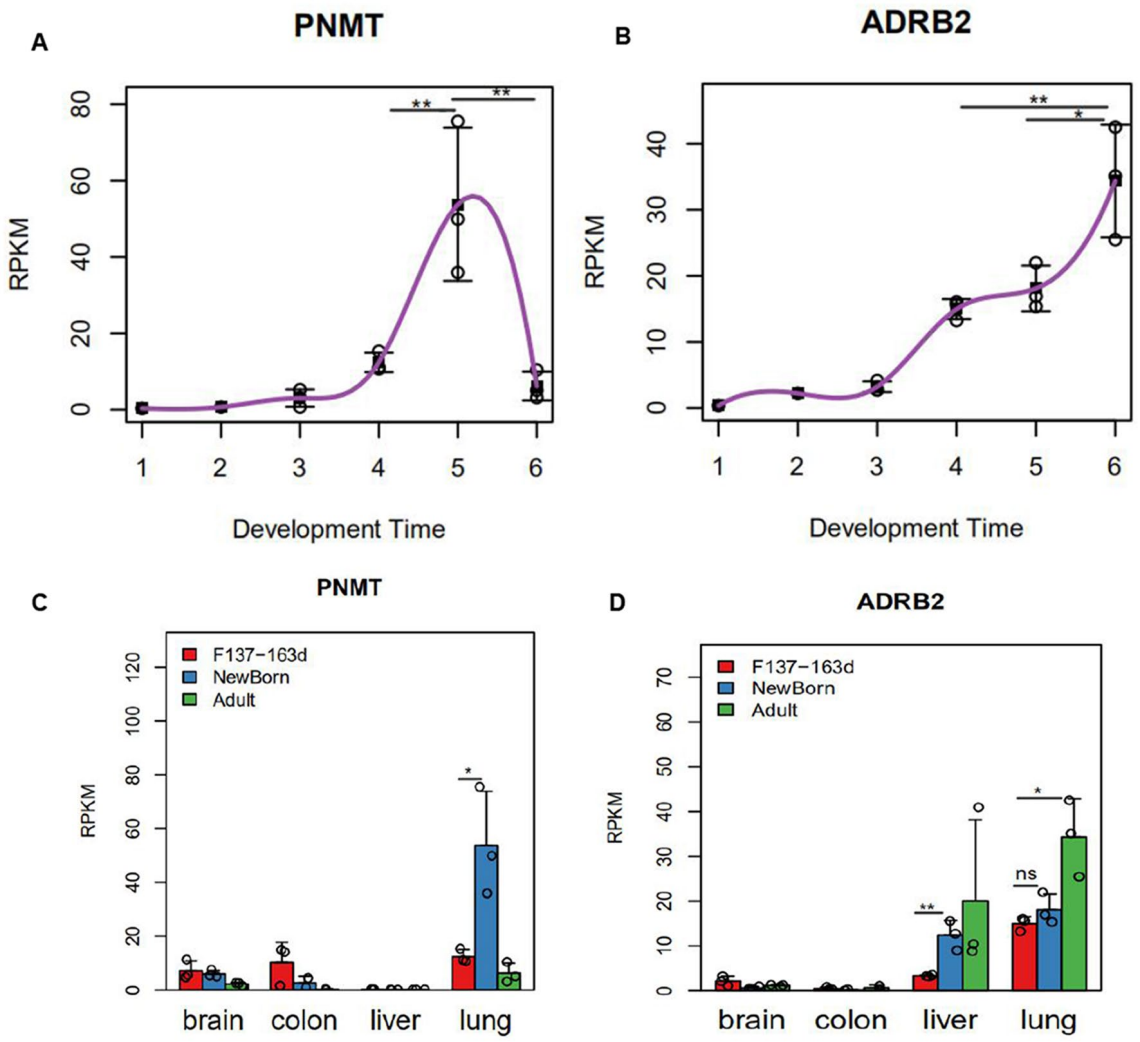
PNMT and ADRB2 expression during lung development in rhesus monkeys. **a**, **b** The Y-axis represents normalized gene expression (RPKM), and the X-axis represents the developmental time point (1 = F45d, 2 = F70d, 3 = F100d, 4 = F137-163d, 5 = Newborn, 6 = Adult). Each point represents a lung sample, and the gray line represents the mean gene expression trend during development. **c**, **d **Comparison of PNMT and ADRB2 expression during lung development in rhesus monkeys as compared with other tissues**.** Gene expression of each group (RPKM, Y-axis) is shown as the mean ± standard error. F137-163d: Days 137 to 163 of pregnancy; Neonate; Days 4, 5, or 7 after delivery; Adult: 5 years or 8 years. During lung development, an independent sample *t* test of the differences in the four organs before and after delivery was performed. *P < 0.05, **p < 0.01

We found that elevated expression of *PNMT* began at the late gestational stage (RPKM > 10) and peaked at the neonatal stage before significantly decreasing at adolescence (Fig. 2a). The temporal spectrum of *ADRB2* expression was generally consistent with that of *PNMT* [i.e., high expression at the late gestational stage (RPKM > 10) and increasing at the neonatal stage]; however, *ADRB2* expression gradually increased with age (Fig. 2b).

### Gene Expression of AC Subtypes

**Fig. 3:**
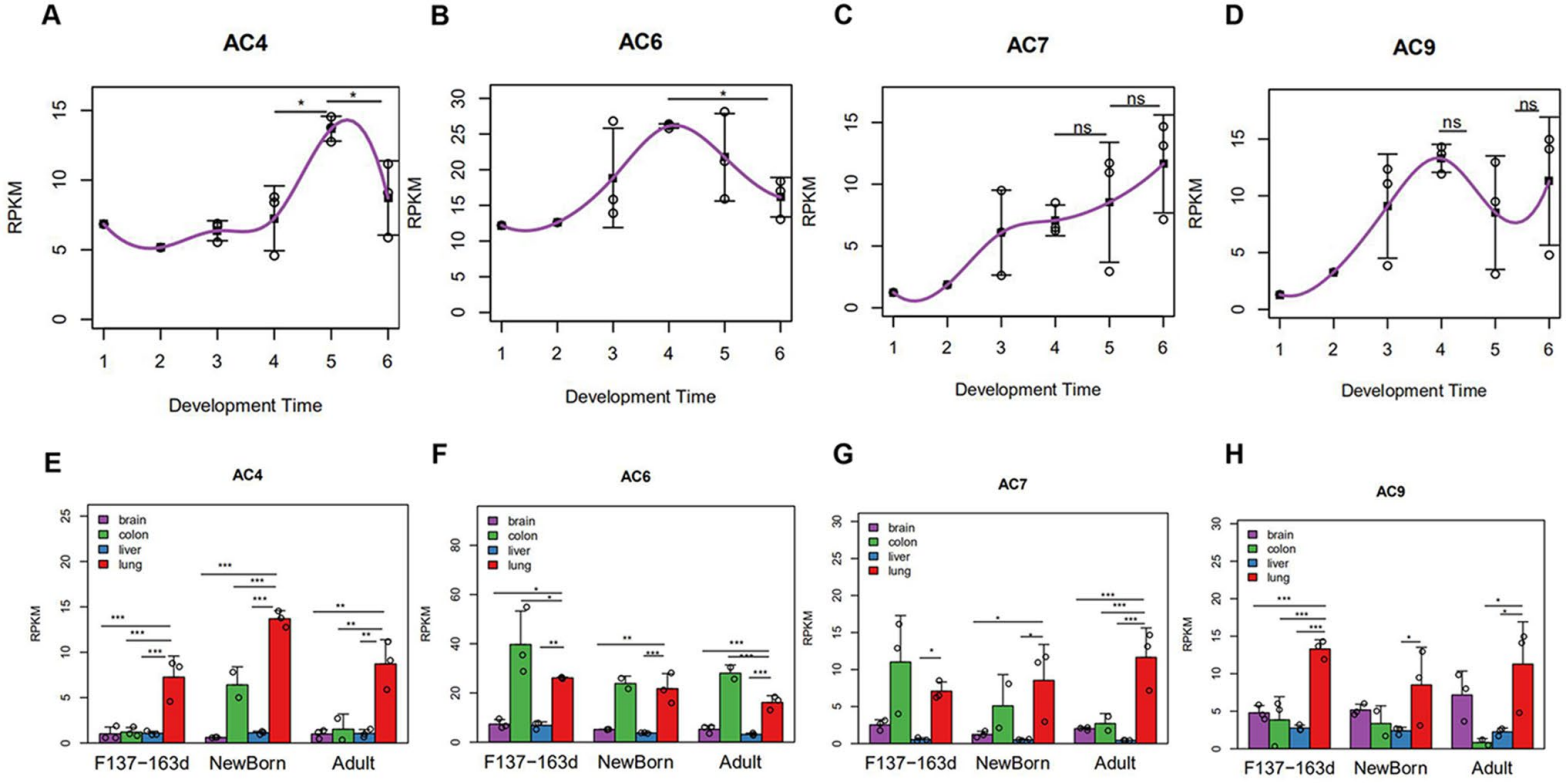
Expressions of some AC subtypes during lung development in rhesus monkeys. **a**–**d** The Y-axis represents normalized gene expression (RPKM), and the X-axis represents the developmental time point (1 = F45d, 2 = F70d, 3 = F100d, 4 = F137-163d, 5 = Newborn, 6 = Adult). Each point represents a lung sample, and the gray line represents the mean gene expression trend during development. **e**–**h **Comparison of expressions of some AC subtypes among four different organs during development in rhesus monkeys. Gene expression of each group (RPKM, Y-axis) is shown as the mean ± standard error. From late gestational stage to adult stage, an independent sample *t* test of the differences in the four organs before and after delivery was performed. *p < 0.05, **p < 0.01, ***p < 0.001

*AC4* expression gradually increased starting at the late gestational stage and peaked at the neonatal stage (Fig. 3a). *AC6* expression was in a downward trend after birth, but remained high throughout the neonatal stage (Fig. 3b). Additionally, *AC7* expression was in a gradually upward trend after birth increased during the entire lung-development process (Fig. 3c). There was no significant difference of the expression of *AC9* among different time points (Fig. 3d).

Comparison of the expression of AC subtypes in the lungs and other organs (brain, intestine, and liver) revealed that *AC4* are mainly expressed in the lungs (Fig. 3e). Moreover, *AC6* displayed the highest expression in the intestine but was also highly expressed in the lungs (Fig. 3f). *AC7* was mainly expressed in lung in adult (Fig. 3g). *AC9* was mainly expressed in the lung at the late gestational stage (Fig. 3h).

### Gene Expression of PDE Subtypes

**Fig. 4:**
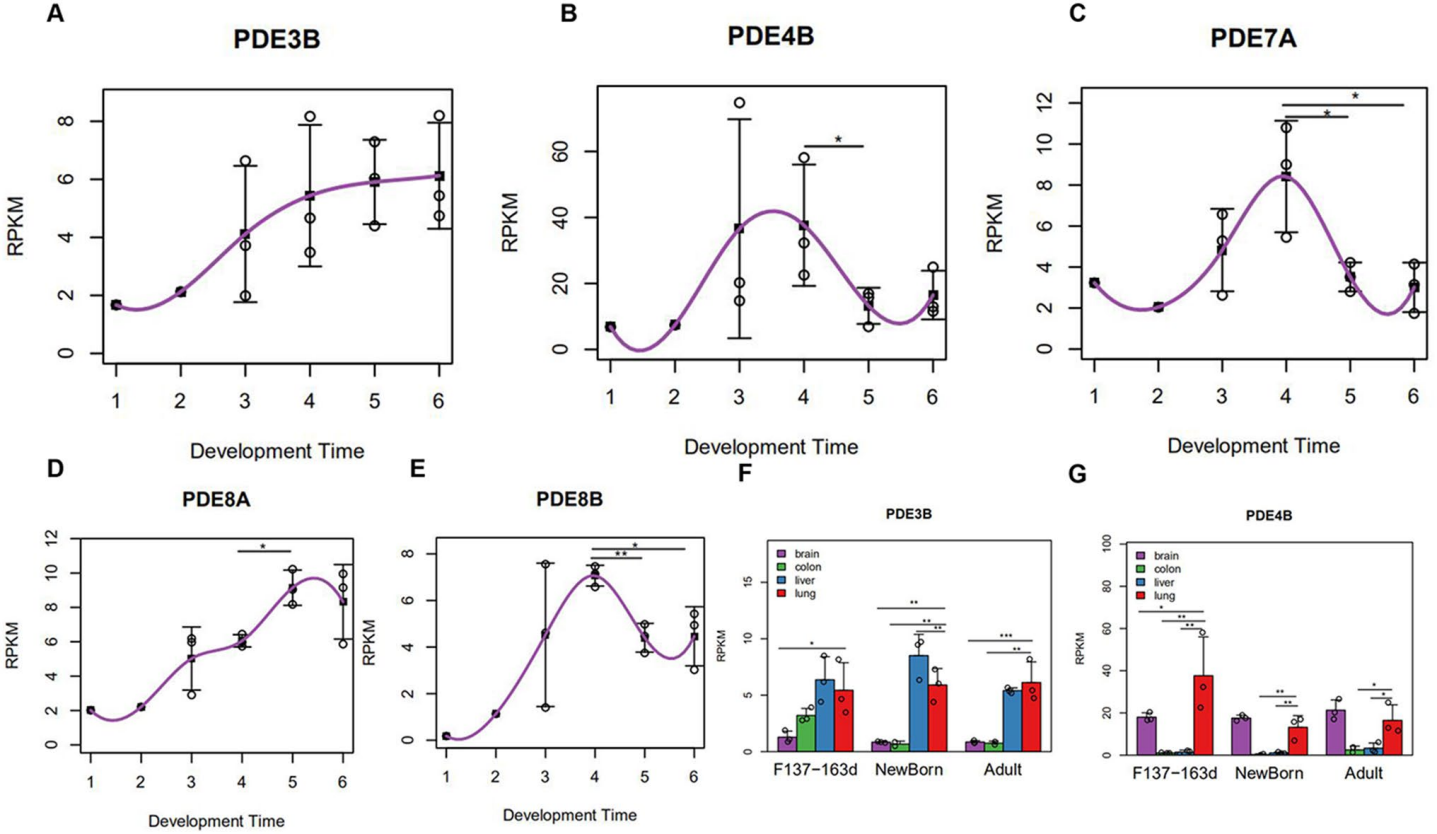
Expressions of some PDE subtypes during lung development in rhesus monkeys. **a**–**e** The Y-axis represents normalized gene expression (RPKM), and the X-axis represents the developmental time point (1 = F45d, 2 = F70d, 3 = F100d, 4 = F137-163d, 5 = Newborn, 6 = Adult). Each point represents a lung sample, and the gray line represents the mean gene expression trend during development. **f**, **g** Comparison of PDE3B and PDE4B expression among four different organs during development in rhesus monkeys. Gene expression of each group (RPKM, Y-axis) is shown as the mean ± standard error. From late gestational stage to adult stage, an independent sample *t* test of the differences in the four organs before and after delivery was performed. *p < 0.05, **p < 0.01, ***p < 0.001

*PDE3B* showed an increasing expression trend in late pregnancy, neonatal period and adulthood (Fig. 4a). *PDE4B* expression peaked at the late gestational stage (Fig. 4b). *PDE7A, PDE7B, PDE8A*, and *PDE8B* expression was persistently low during the entire lung-developmental process (RPKM > 1, Fig. 1), although *PDE7A* and *PDE8B* expression was highest at the late gestational stage (Fig. 4c, e). *PDE8A* expression increased during gestational stages and decreased significantly at birth (Fig. 4d).

*PDE3B* was mainly expressed in the lung as well as in the liver (Fig. 4f), and there was no significant difference of *PDE4B* expression between lung and brain after birth (Fig. 4g).

### Gene Expression of AKAP and EPAC Subtypes

**Fig. 5:**
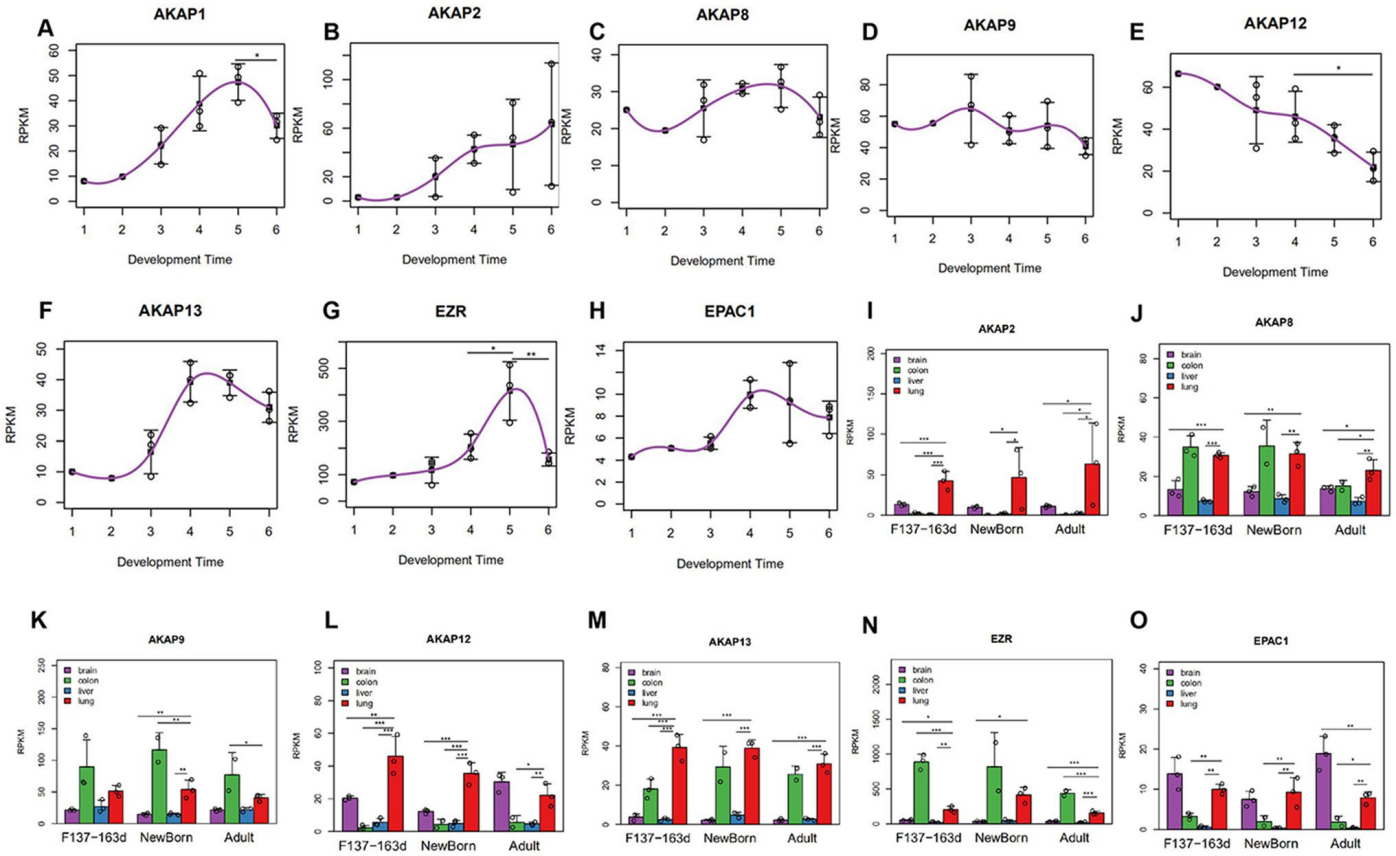
Expressions of some AKAP and EPAC subtypes during lung development in rhesus monkeys. **a**–**h** The Y-axis represents normalized gene expression (RPKM), and the X-axis represents the developmental time point (1 = F45d, 2 = F70d, 3 = F100d, 4 = F137-163d, 5 = Newborn, 6 = Adult). Each point represents a lung sample, and the gray line represents the mean gene expression trend during development. **i**–**o** Comparison of some AKAP and EPAC subtypes expression among four different organs during development in rhesus monkeys. Gene expression of each group (RPKM, Y-axis) is shown as the mean ± standard error. From late gestational stage to adult stage, an independent sample *t* test of the differences in the four organs before and after delivery was performed. *p < 0.05, **p < 0.01, ***p < 0.001

*AKAP1* expression peaked at birth (Fig. 5a). *AKAP2* expression was in an increasing trend during all developmental stages (Fig. 5b). *AKAP(8/9/13)*and *EPAC1* expression were stable during all developmental stages (Fig. 5c, d, f, and h). *AKAP12* expression decreased continuously during all developmental stages (Fig. 5e). *EZR* expression peaked in neonatal period (Fig. 5g).

Compared with the expression pattern of 18 genes in the brain, colon, and liver, *AKAP*(*2/8*) was mainly expressed in the lung in adulthood (Fig. 5i, j). In the neonatal period, *AKAP*(*8/9/13*) were mainly expressed in the lung in addition to the intestinal tract (Fig. 5j, k, m). *AKAP12* was mainly expressed in the lungs of newborns, and was also highly expressed in the lung and the brain in adults (Fig. 5l). *EZR* was mainly expressed in the lung and intestinal tract (Fig. 5n). *EPAC1* was mainly expressed in the brain and lung (Fig. 5o).

## Discussion

PNMT is a rate-limiting and essential enzyme that catalyzes the methylation of noradrenaline to adrenaline [16], and the only N-methyltransferase that can synthesize adrenaline [17]. Human PNMT is mainly expressed in the adrenal medulla and also present in human lung tissues [17, 18]. Previous studies report that the lungs can synthesize adrenaline locally and regulate adrenaline. Moreover, PNMT in human lungs and bronchial epithelial cells exhibit high substrate affinity and specificity similar to that of adrenal PNMT [17]. In the present study, we found persistent high expression of *PNMT* at the late gestational and neonatal stages in rhesus monkeys, with its peak observed at the neonatal stage. Moreover, *PNMT* expression in the lungs at the neonatal stage was significantly higher than that in other tissues (brain, intestine, and liver), suggesting that the respiratory tract is connected to the external environment after birth, and that various natural stimuli require dynamic adaptations (such as airway relaxation). Adrenaline expression is an important method of bodily adaptation; therefore, *PNMT* expression might represent a preparatory mechanism for increasing adrenaline. These findings suggest that a role of elevated *PNMT* expression might be to prepare the fetus for birth maintaining its health at the neonatal stage.

Both *ADRB2* and *PNMT* expression showed persistent and progressive increases in the late gestational and neonatal stages; however, in contrast to *PNMT*, *ADRB2* expression increased with age and showed differences in expression only in the liver before and after delivery. Moreover, *ADRB2* expression in the lungs at the neonatal stage was not significantly different, whereas significant differences were present at the adolescence stage. This confirms that the density of ADRB2 receptor is related to age, reaching adult levels at school age [19]. ADRB2 is mainly located in airway smooth muscle (ASM) cells, type II pneumocytes, mast cells, small blood vessels in the bronchi, and epithelial cells, among which ASM cell density is the highest. The main function of ADRB2 in ASM cells is to relax the airway [4]. These findings suggest that PNMT and ADRB2 activate the Gs–AC–PKA signaling pathway to cause airway dilation, thereby ensuring optimal ventilation in the lungs [20]. Furthermore, we found elevated expression of both PNMT and ADRB2 in the late gestational stage; therefore, we speculate that this adrenergic mechanism also applies to premature infants.

The results showed that all subtypes (except *AC8, AKAP4/5*) associated with the ADRB2–Gs–AC signaling pathway were expressed in the lungs at the neonatal stage. These results indicated that this signaling pathway might play a role in airway dilation during the neonatal stage.

We speculated that AC6 might play an important role in this signaling pathway. Previous studies report that transcripts of all AC subtypes, except *AC2*, are detected in human ASM, and western blot results and functional testing show that AC5/6 exhibit important functions in hASM [21]. Xu et al. detected mRNA for three AC subtypes (*AC2*, *AC4*, and *AC6*) in cultured hASM cells [22], and Shailesh et al. reported that the ADRB2 response in hASM is mainly associated with AC6 in lipid rafts [23]. These findings indicated that specific expression of AC subtypes in hASM remains unclear.

PDE3 and PDE4 are the two major cAMP-hydrolyzing enzymes in ASM. PDE3 is an enzyme hydrolyzing both cAMP and cGMP, but the rate of hydrolyzing cAMP is 10 times that of hydrolyzing cGMP. PDE3 and PDE4 can regulate different cAMP pools because they are located in different parts of the ASM [24, 25]. PDE3 is located in a compartment more closely associated to regulation of Ca^2+^ fluxes affecting contractility. PDE3 inhibitor is important in preventing mast cells predominantly in the ASM layer degranulation. Therefore, it is an acute bronchodilator in humans [26–29]. However, PDE4 inhibitor cannot induce acute bronchodilator, which is consistent with its lack of mast cell or ASM function [24, 28]. Despite this, PDE4 inhibitors have been found to reduce the pro-inflammatory activity of hASM cells and thus increase airway relaxation, and PDE4 inhibitors show some efficacy against the late asthmatic response [30–32]. Some scholars have proposed that PDE3/4 combined inhibitors have better bronchodilation and anti-inflammatory activities [29, 33]. In this paper, *PDE3* and *PDE4* were expressed during the entire lung-development process. We speculate that the application of double PDE3/4 inhibitors may be feasible. Moreover, we found that *PDE4B* was highly expressed during the entire lung-development process. Previous studies proposed that PDE4B and PDE4D played critical roles in airway cells [34, 35], PDE4B performed many beneficial anti-inflammatory effects without the side effects, whereas PDE4D had vomiting effects related to central nervous system (CNS) [36, 37]. We should pay more attention to PDE4B in ASM. However, PDE4B is also highly expressed in brain tissues and might be involved in CNS-related side effects.

AKAP has been shown to regulate intracellular cAMP localization and regulate ADRB2 signaling in human ASM [38]. We found that *AKAP*(1/2/*8/9/12/13,* and *EZR*) were highly expressed during the entire lung-development process. Previous studies have shown that there are protein and/or mRNA expressions of *AKAP*(*1/2/3/5/8/9/10/11/12/13*, *EZR*, and *MAP2B*) subtypes in human ASM. Especially, *AKAP12* and *EZR* were highly expressed [8, 39]. EZR(also known as Ezrin) is considered to be a key regulator of airway membrane receptor complex and its signal transduction pathway [40]. We speculate that AKAP(1/2/*8/9/12/13,* and EZR) play an important role in this pathway, especially EZR. Further studies are needed to confirm the expression of AKAPs subtypes in ASM.

However, our study has some limitations. First, because our samples were limited, we did not perform reverse transcription polymerase chain reaction analysis to confirm expression levels, western blotting analysis to detect protein levels, or immunohistochemical analysis to determine protein localization. We focused solely on the expression of key genes in this signaling pathway in the entire lung and did not measure the expression and activity in specific cell types in ASM; therefore, this requires further study. Second, the sample size of rhesus monkeys in this study was low. Moreover, there might be species-specific differences in the expression of various subtypes. Therefore, it remains unclear whether these results truly reflect human lung development.

## Conclusion

We compared the expression levels of key genes associated with the ADRB2 signaling pathway at different developmental stages in the lungs of rhesus monkeys. We found that almost all key genes of this classical signaling pathway are expressed at the neonatal stage, which provides references for correct application of therapeutics for diseases associated with this signaling pathway in neonates. Furthermore, our findings that the expression of specific subtypes dominated during lung development provide novel insights that will promote the development of novel strategies for treating respiratory diseases.
